# SEQUOIA: significance enhanced network querying through context-sensitive random walk and minimization of network conductance

**DOI:** 10.1186/s12918-017-0404-6

**Published:** 2017-03-14

**Authors:** Hyundoo Jeong, Byung-Jun Yoon

**Affiliations:** Department of Electrical and Computer Engineering, Texas A&M University, College Station, TX USA

## Abstract

**Background:**

Network querying algorithms provide computational means to identify conserved network modules in large-scale biological networks that are similar to known functional modules, such as pathways or molecular complexes. Two main challenges for network querying algorithms are the high computational complexity of detecting potential isomorphism between the query and the target graphs and ensuring the biological significance of the query results.

**Results:**

In this paper, we propose SEQUOIA, a novel network querying algorithm that effectively addresses these issues by utilizing a context-sensitive random walk (CSRW) model for network comparison and minimizing the network conductance of potential matches in the target network. The CSRW model, inspired by the pair hidden Markov model (pair-HMM) that has been widely used for sequence comparison and alignment, can accurately assess the node-to-node correspondence between different graphs by accounting for node insertions and deletions. The proposed algorithm identifies high-scoring network regions based on the CSRW scores, which are subsequently extended by maximally reducing the network conductance of the identified subnetworks.

**Conclusions:**

Performance assessment based on real PPI networks and known molecular complexes show that SEQUOIA outperforms existing methods and clearly enhances the biological significance of the query results. The source code and datasets can be downloaded from http://www.ece.tamu.edu/~bjyoon/SEQUOIA.

**Electronic supplementary material:**

The online version of this article (doi:10.1186/s12918-017-0404-6) contains supplementary material, which is available to authorized users.

## Background

Protein-protein interaction (PPI) plays pivotal roles in understanding biological systems. Diverse functional modules in cells, such as signaling pathways and protein complexes, involve numerous proteins and their functions are governed by the intertwined interactions among these proteins. For this reason, to better understand the functions and roles of proteins in cells, it is critically important to investigate how groups of proteins collaborate with each other to perform certain biological functions and achieve common goals, in addition to studying the functions of individual proteins. Recent advances in technologies for high throughput measurement of protein-protein interactions have enabled genome-scale studies of protein interactions, and systematic analyses of the available PPI networks may reveal new functional network modules and unveil novel functionalities of the proteins that are involved in such modules. Recent investigations of PPI networks show that functionally important network modules (e.g., molecular complexes and pathways) are often well conserved across networks of different species [1, 2]. These observations clearly point to comparative network analysis [3] as a promising solution for effectively analyzing large-scale PPI networks, detecting common functional modules that are embedded in the networks, and predicting the functions of proteins that comprise these modules.

Network querying is one possible way of comparatively analyzing biological networks, which can be especially useful when prior knowledge of functional modules is available for a given species. As implied in its name, network querying aims to find out whether a target network (typically, belonging to another species) contains network modules that resemble the module that is being used as the query [3]. This provides an efficient way of transferring knowledge between species, since we could use computational means to predict potential network modules in a new (or less-studied) species that may have similar functions, structures, and underlying mechanisms to well-studied modules in other species.

Several network querying algorithms have been proposed so far [4–10]. PathBLAST [4] has been designed to identify conserved signaling pathways. However, it can only handle linear pathways and its high computational complexity places a stringent restriction on the maximum length of the pathway that could be searched. QPath [5] can search for longer pathways and QNet [6] can search for linear pathways as well as trees, but both algorithms are not suitable for large queries due to their high computational cost. To overcome restrictions on the topology of the query network, several network querying algorithms have been proposed that can identify network modules with arbitrary topology [7–10]. For example, TORQUE [7] finds a connected subnetwork of matching proteins in the target network based on sequence similarity, without explicitly utilizing the topological structure of the query network in identifying conserved functional modules. NatalieQ [10] formulates the network alignment problem as an integer linear programming problem, and solves the optimization problem using Lagrangian relaxation combined with a branch-and-bound approach. RESQUE [8] adopts a semi-Markov random walk (SMRW) model to estimate the node correspondence between the query and the target networks, based on which it iteratively reduces the target network by removing irrelevant nodes. Once the target network has been sufficiently reduced, RESQUE identifies the best matching subnetwork either by the Hungarian method or by identifying the largest connected subnetwork. Another recent algorithm, called Corbi [9], measures the node correspondence between networks based on a conditional random field (CRF), after which the matching subnetwork is identified through an iterative bi-directional mapping.

Most of the aforementioned network querying methods consider both *node similarity* and *topological similarity* between the query and the target networks to detect matching subnetworks in the target network. Node similarity between nodes that belong to different networks is typically measured based on sequence similarity. Topological similarity between (sub)networks are measured in various ways to capture the molecular interaction patterns that are conserved across networks. Incorporating both types of similarities has been shown to be crucial in making biologically relevant predictions about conserved functional modules [1–3, 11]. However, one important aspect of network module detection that is often neglected in network querying is that such modules are often well separated from the rest of the network. In fact, this separability has played critical roles in “non-comparative” network analysis methods that aim to detect modules or sub-communities in a given network [12–14], since molecules in a functional module tend to be densely connected to other molecules in the same module but loosely connected to nodes that are not part of the module. Although identifying densely connected subnetwork modules is not the main objective of network querying, explicitly incorporating separability criterion into comparative network analysis methods has strong potentials to enhance the quality of the predictions [15].

In this paper, we propose a novel network querying algorithm called SEQUOIA (Significance Enhanced QUerying Of InterAction networks). The proposed algorithm is built on the following important concepts: (i) effective estimation of *node correspondence* – or overall functional similarity between nodes in different networks – by sensibly combining sequence similarity and interaction pattern similarity through a random walk model; and (ii) minimization of network conductance of potential network modules, thereby identifying matching modules in the target network that are well separated from the rest of the network. In our proposed algorithm, we first estimate the node correspondence based on a context-sensitive random walk model [16, 17], and select a seed network based on the estimated node correspondence scores. Then, the seed network is iteratively extended by adding the nodes that maximally reduce the conductance of the subnetwork. Finally, the significance enhanced querying result is achieved by keeping the nodes with acceptable extension reward scores, which are updated for every node at each extension step. Through extensive evaluations based on real biological complexes, we show that SEQUOIA can remarkably enhance the biological significance of the network querying results by estimating the node correspondence based on the CSRW model and minimizing the conductance of matching network modules.

## Methods

### Problem formulation and overview of the proposed method

Suppose that we have a query protein-protein interaction (PPI) network represented by a graph $\mathcal {G}_{\mathcal {Q}} = \left (\mathcal {V}_{\mathcal {Q}},\mathcal {E}_{\mathcal {Q}} \right)$, which has a set of nodes $\mathcal {V}_{\mathcal {Q}} = \left \{ v_{1}, v_{2}, \ldots \right \}$ and set of edges $\mathcal {E}_{\mathcal {Q}} = \left \{ {e_{i,j}} \right \}$. A protein in the query network is represented as a node ${v_{i}} \in \mathcal {V}_{\mathcal {Q}}$ in the graph $\mathcal {G}_{\mathcal {Q}}$ and the interaction between two proteins *v*_*i*_ and *v*_*j*_ is represented by an edge *e*_*i*,*j*_, whose weight *w*_*i*,*j*_ reflects the strength (or confidence) of the interaction. Similarly, suppose we are also given a target PPI network represented by a graph $\mathcal {G}_{\mathcal {T}} = \left (\mathcal {V}_{\mathcal {T}},\mathcal {E}_{\mathcal {T}} \right)$. We define the size of a network as the number of nodes in the given network, hence the size of the query network is $\left | \mathcal {V}_{\mathcal {Q}} \right |$ and that of the target network is $\left | \mathcal {V}_{\mathcal {T}}\right |$. Typically, in a network querying problem, the size of the target network is significantly larger than the query network (i.e., $\left | \mathcal {V}_{\mathcal {Q}} \right | \ll \left | \mathcal {V}_{\mathcal {T}} \right |$). We assume that a pairwise node similarity score *s*(*v*_*q*_,*v*_*t*_) is available $\forall {v_{q}} \in \mathcal {V}_{\mathcal {Q}}$ and $\forall {v_{t}} \in \mathcal {V}_{\mathcal {T}}$, reflecting the molecular level similarity between the proteins in the query network and the target PPI network. In this study, we use the BLAST bit score as the pairwise node similarity score as in most network querying and alignment algorithms.

The main objective of network querying is to find the conserved subnetwork $\hat {\mathcal {G}}_{\mathcal {T}} = \left (\hat {\mathcal {V}}_{\mathcal {T}},\hat {\mathcal {E}}_{\mathcal {T}} \right)$ within the target PPI network $\mathcal {G}_{\mathcal {T}} = \left (\mathcal {V}_{\mathcal {T}},\mathcal {E}_{\mathcal {T}} \right)$ that bears the largest overall functional similarity to the given query network $\mathcal {G}_{\mathcal {Q}} $. Therefore, we can formulate the network querying problem as the following optimization problem:

1 $$ \hat{\mathcal{G}}_{\mathcal{T}}^{*} = \mathop {\arg \max }\limits_{\hat {\mathcal{G}}_{\mathcal{T}} \in {\mathbf{G}}_{\mathbf{T}}} f\left({\hat {\mathcal{G}}_{\mathcal{T}},{\mathcal{G}}_{\mathcal{Q}}} \right),   $$

where **G**_**T**_ is the set of all possible subnetworks of the target PPI network, and $f\left ({\mathcal {G}}_{x},{\mathcal {G}}_{y} \right)$ is a function that measures the overall functional similarity between two networks ${\mathcal {G}}_{x}$ and ${\mathcal {G}}_{y}$.

The network querying problem can be reformulated as a subgraph isomorphism problem, whose goal is to find a bijection between two graphs. In order to find a one-to-one mapping, deleted nodes can be modeled as dummy nodes so that an inserted node in the query network can be mapped to a dummy node in the target network, and vice versa. The subgraph isomorphism problem is known to be NP-complete [18], hence the existence of a polynomial time algorithm for solving the problem is unknown. Furthermore, it is also not straightforward to quantitatively estimate the overall functional similarity $f\left ({\mathcal {G}}_{x},{\mathcal {G}}_{y} \right)$ between two networks ${\mathcal {G}}_{x}$ and ${\mathcal {G}}_{y}$ in such a way that is biologically meaningful. As a result, it is practically challenging to effectively formulate the optimization problem in (1) and solve the problem for large-scale networks in a computationally efficient manner [6–8]. A reasonable way to estimate this functional similarity is to define $f\left ({\mathcal {G}}_{x},{\mathcal {G}}_{y} \right)$ by sensibly combining the node similarity and the topological similarity between the networks under comparison [3]. Given a reasonable $f\left ({\mathcal {G}}_{x},{\mathcal {G}}_{y} \right)$, heuristic optimization schemes may have to be employed to make the optimization problem (1) computationally tractable.

In our proposed network querying algorithm SEQUOIA, we first pre-process the target network by removing non-homologous nodes and inserting pseudo-edges between nodes that are likely to share similar functionalities. Next, the query and the target networks are compared and node correspondence scores are estimated using the context-sensitive random walk (CSRW) model [16]. The resulting scores are used to select a “seed network” that consists of target nodes that have strong correspondence to query nodes. The seed network is extended by iteratively adding the nodes that maximally reduce the network conductance of the extended network, through which SEQUOIA aims to find a subnetwork that is densely connected within the subnetwork while sparsely connected to the rest of the target network. This has the effect of identifying a subnetwork in the target PPI network that closely matches the query, and at the same time, has strong potential to be a functional network module. Finally, the extended subnetwork is pruned by removing potentially irrelevant nodes that contribute little to making the network dense, which improves the functional coherence of the querying results, as will be demonstrated later.

### The context-sensitive random walk (CSRW) model

**Fig. 1:**
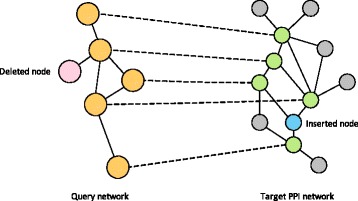
Illustration of network querying, which aims to identify the subnetwork region in the target network that best matches the given query. *Gray colored nodes* in the target network are irrelevant to the query network. In the example shown, the *pink colored node* in the query network is deleted in the target network, while the *blue colored node* is inserted in the target

Here, we briefly review the CSRW model [16] that is used for estimating the correspondence between nodes in the query and the target networks. To accurately estimate the node correspondence, it is desirable to effectively integrate the node similarity (sequence similarity between proteins) and topological similarity (similarity between interaction patterns for different proteins), as mentioned previously. However, as depicted in Fig. 1, inserted and deleted nodes in the conserved network can make effective estimation of the node correspondence difficult. The CSRW model has been recently proposed to explicitly model such node insertions and deletions, while integrating the two types of similarities to compute the node correspondence scores.

At a given moment, the random walker in the CSRW model is located at a node pair (*v*_*q*_,*v*_*t*_), where *v*_*q*_ is a node in the query network and *v*_*t*_ is a node in the target network. At each step, the walker makes a random move to neighboring nodes, where it switches between two different types of modes of random walk – namely, *simultaneous walk* on both networks and *individual walk* on one of the networks – depending on the surrounding “context” of the walker’s current location. For example, if *v*_*q*_ and *v*_*t*_ have neighboring nodes with positive node similarity, the random walker simultaneously moves on both networks to such nodes. Otherwise, it randomly selects one of the networks and randomly moves only on the selected network. Further details of the CSRW model can be found in the supplementary material (see Additional file 1: Section S1). In the long run, the random walker is designed to simultaneously visit node pairs with better correspondence (i.e., with higher node similarity and topological similarity) more frequently. Based on the design, the long-run proportion of time that the random walker simultaneously visits a given pair of nodes can be used as a probabilistic measure of the correspondence between the nodes [16]. This long-run proportion of time, or the steady-state probability of the CSRW model, can be efficiently computed in practice using the power method, as real PPI networks tend to be very sparse [8, 19]. We use the steady-state probability of the context-sensitive random walker as the node correspondence score $c\left ({v_{q},v_{t}} \right),\forall v_{q} \in {\mathcal {V}}_{\mathcal {Q}}$ and $\forall v_{t} \in {\mathcal {V}}_{\mathcal {T}}$, and the node correspondence scores for all node pairs can be concisely written in a $\left | {{\mathcal {V}}_{\mathcal {Q}}} \right | \times \left | {{\mathcal {V}}_{\mathcal {T}}} \right |$ dimensional matrix **C**. The context-awareness of the CSRW model makes it robust to potential node insertions/deletions, and the model has been shown to be useful for estimating node correspondence [16]. In fact, the CSRW-based node correspondence scores have been recently applied to multiple network alignment [17], where they have been shown to clearly enhance the overall alignment accuracy.

### SEQUOIA network querying algorithm

Before computing the node correspondence scores based on the CSRW model, we perform two pre-processing steps. First, we reduce the target network by removing potential non-homologous nodes. Specifically, we remove every node *v*_*t*_ in the target network whose node similarity *s*(*v*_*q*_,*v*_*t*_) never exceeds a given threshold *T*_*h*_ for any of the query nodes $v_{q} \in {\mathcal {V}}_{\mathcal {Q}}$. In this study, we set the threshold *T*_*h*_ as 0, such that a node is kept in the target network if it has at least one query node with nonzero similarity score. Removing target nodes that do not have any homologous node in the query network can significantly reduce the computation time as well as the memory requirement. Second, since removing non-homologous nodes may make the target network disconnected, we insert a pseudo-edge between nodes that are likely to share similar functionalities, motivated by the fact that proteins with direct interactions are more likely to share similar functionalities [20]. For this purpose, we assumed that any two nodes in the target network are likely to share similar functionalities and may potentially have a direct interaction if they have a common node in the query network with high node similarity. However, to refrain from inserting too many false-positive pseudo edges, we only insert a pseudo edge if the two nodes under consideration belong to different subnetworks that are disconnected from each other. Since current PPI networks are incomplete and noisy – with many false positive interactions as well as false negative interactions [21, 22] – adding pseudo-edges to the reduced target network can lead to more reliable querying results, as will be demonstrated in our simulation results. Further details of the pre-processing step can be found in the supplementary material (see Additional file 1: Section S2) with an illustrative example.

After pre-processing the target network, the CSRW model is used to estimate the correspondence between nodes in the query and the target networks. The resulting node correspondence score matrix **C** is normalized to obtain the normalized score matrix $\bar {\mathbf {C}}$ using the normalization method proposed in [19]:

2 $$ \bar{\mathbf{C}} = \frac{1}{2}\left[ {{\mathbf{J}}_{\mathbf{L}} \cdot {\mathbf{C}} + {\mathbf{C}} \cdot {\mathbf{J}}_{\mathbf{R}}} \right].  $$

The matrix $\bar {\mathbf {C}}$ is a $\left | {{\mathcal {V}}_{\mathcal {Q}}} \right | \times \left | {{\mathcal {V}}_{\mathcal {T}}} \right |$ dimensional matrix containing the normalized node correspondence scores, **J**_**L**_ is a $\left | {{\mathcal {V}}_{\mathcal {Q}}} \right | \times \left | {{\mathcal {V}}_{\mathcal {Q}}} \right |$ dimensional diagonal matrix with the diagonal term ${\mathbf {J}}_{\mathbf {L}} \left ({q,q} \right) = {1 \left / {{\sum \nolimits }_{t = 1}^{\left | {{\mathcal {V}}_{\mathcal {T}}} \right |} {c\left ({v_{q},v_{t}} \right)} } \right.}$, and **J**_**R**_ is a $\left | {{\mathcal {V}}_{\mathcal {T}}} \right | \times \left | {{\mathcal {V}}_{\mathcal {T}}} \right |$ dimensional diagonal matrix with the diagonal term ${\mathbf {J}}_{\mathbf {R}} \left ({t,t} \right) = {1 \left / {{\sum \nolimits }_{q = 1}^{\left | {{\mathcal {V}}_{\mathcal {Q}}} \right |} {c\left ({v_{q},v_{t}} \right)} } \right.}$. This normalization step aims to estimate the *relative* significance between corresponding nodes, which has been shown to be useful for comparing networks of different size [19]. Based on the normalized correspondence score $\bar {\mathbf {C}}$, we iteratively select *N*_*Q*_ seed nodes in the target network based on the following rule:

3 $$ {\mathop {\arg \min }\limits_{v_{t}} \left[ {\prod\limits_{v_{q} \in {\mathcal{V}}_{\mathcal{Q}}} {\left({1 - \bar c\left({v_{q},v_{t}} \right)} \right)}} \right]}.  $$

The above selection rule aims to identify the nodes in the target network that have a large number of highly corresponding nodes in the query network. The score $\bar c\left ({v_{q},v_{t}} \right)$ will be close to 1 for a highly corresponding node pair (*v*_*q*_,*v*_*t*_). Therefore, the product $\prod _{v_{q} \in {\mathcal {V}}_{\mathcal {Q}}} {\left ({1 - \bar c\left ({v_{q},v_{t}} \right)} \right)}$ will approach 0 for a target node *v*_*t*_ (i.e., a potential seed node) that has a large number of query nodes $v_{q} \in {\mathcal {V}}_{\mathcal {Q}} $ with a high node correspondence score $\bar c\left ({v_{q},v_{t}} \right)$. This is based on an assumption that a target node with a larger number of relevant nodes in the query network may be more likely to be involved in similar functions as the query network compared to a node that has fewer corresponding nodes. After selecting the *N*_*Q*_ seeds, we find the largest connected subnetwork based on the *N*_*Q*_ seed nodes, which is referred to as the seed network. In this work, we set $N_{Q} = \left | {{\mathcal {V}}_{\mathcal {Q}}} \right |$ so that the size of the seed network does not exceed the size of the query network.

Once the seed network is obtained, we iteratively extend the network by adding nodes that can make the extended network well-separated from the rest of the network. To this aim, we estimate the conductance of the subnetwork and define the extension reward score for each node as follows. First, given a network ${\mathcal {G}} = \left ({{\mathcal {V}}_{\mathcal {G}},{\mathcal {E}}_{\mathcal {G}}} \right)$, suppose that we have a Gaussian surface enclosing the subnetwork ${\mathcal {H}} = \left ({{\mathcal {V}}_{\mathcal {H}},{\mathcal {E}}_{\mathcal {H}}} \right)$ such that ⊆. Then, the conductance *φ* of the subnetwork ${\mathcal {H}}$ is defined as the number of edges that pass through the surface divided by the volume of the subnetwork (i.e., the number of edges that are enclosed by the surface) [23, 24]. The conductance of the subnetwork $\mathcal {H}$ is given by

4 $$ \phi \left({\mathcal{H}} \right) = \frac{{\left| {\left\{ {e_{i,j} |i \in {\mathcal{V}}_{\mathcal{H}},j \in {\mathcal{V}}_{\bar {\mathcal{H}}}} \right\}} \right|}}{{\min \left({vol\left({{\mathcal{V}}_{\mathcal{H}}} \right),vol\left({{\mathcal{V}}_{\bar {\mathcal{H}}}} \right)} \right)}},  $$

where $\bar {\mathcal {H}} = \left ({{\mathcal {V}}_{\mathcal {G}} \backslash {\mathcal {V}}_{\mathcal {H}},{\mathcal {E}}_{\mathcal {G}} \backslash {\mathcal {E}}_{\mathcal {H}}} \right)$, and $vol\left ({{\mathcal {V}}_{\mathcal {X}}} \right) = \sum \limits _{u \in {{\mathcal {V}}_{\mathcal {X}} }} {d\left (u \right)}$, where *d*(*u*) is the degree of the node *u*. In a network querying problem, since the conserved subnetwork is typically significantly smaller than the rest of the target PPI network, the volume of the querying result is also much smaller than the volume of the rest of the target network, i.e., $vol\left ({{\mathcal {V}}_{\mathcal {H}}} \right) \ll vol\left ({{\mathcal {V}}_{\bar {\mathcal {H}}}} \right)$. Hence, the conductance of the subnetwork ${\mathcal {H}}$ becomes

5 $$ {}\begin{aligned} \phi \left({\mathcal{H}} \right) = \frac{{\left| {\left\{ {e_{i,j} |i \in {\mathcal{V}}_{\mathcal{H}},j \in {\mathcal{V}}_{\bar {\mathcal{H}}}} \right\}} \right|}}{{vol\left({{\mathcal{V}}_{\mathcal{H}}} \right)}} = \frac{{\left| {\left\{ {e_{i,j} |i \in {\mathcal{V}}_{\mathcal{H}},j \in {\mathcal{V}}_{\bar {\mathcal{H}}}} \right\}} \right|}}{{\left| {\left\{ {e_{i,j} |i,j \in {\mathcal{V}}_{\mathcal{H}}} \right\}} \right|}}. \end{aligned}  $$

Second, we define the extension reward score for a given node as the number of newly added neighboring nodes during the extension step. That is, in each extension step, when we add a new node, all neighboring nodes in the extended subnetwork will get an extra extension reward score of 1. Based on the extension reward score, we can measure the contribution of each node towards making the subnetwork dense. A node with a higher extension reward score interacts with a larger number of newly added nodes, playing a more significant role in making the subnetwork dense after adding the new nodes.

In each extension step, we add the node which is densely connected to the nodes within the extending network and loosely connected to the nodes out of the extending network, in order to minimize the conductance defined in (5). We repeat the extension steps until there is no more neighboring node that can reduce the current conductance by more than 5 percent or until the size of extending network exceeds twice the size of the query network, whichever occurs first. Once the extension process comes to an end, we remove all nodes whose extension reward score does not exceed a certain threshold. This is to enhance the functional coherence of the final querying result, since nodes with fewer interactions are relatively less likely to share similar functionalities with other neighbors. However, the original seed nodes are kept in the final result, even if their extension reward score is not large, since those nodes have high node correspondence to nodes in the query network. In this study, we set the threshold for node removal as 0, so that nodes that do not interact with any of the newly added nodes are removed in the final querying result. The overall procedure of the proposed SEQUOIA network querying algorithm is summarized in Algorithm 1.

## Results and discussion

### Datasets and experimental set-up

To assess the performance of SEQUOIA, we carried out network querying experiments based on the real PPI networks of three different species – *H. sapiens* (human), *S. cerevisiae* (yeast), and *D. melanogaster* (fly) – obtained from [25]. PPI networks in [25] were originally obtained from the STRING database [26], but interactions between proteins without experimental validation were removed. The human PPI network contains 12,575 proteins and 86,890 interactions, the fly PPI network contains 8624 proteins and 39,466 interactions, and the yeast PPI network contains 6136 proteins and 166,229 interactions.

As the query networks, we used protein complexes obtained from [7], comprised of complexes in three species: *H. sapiens*, *S. cerevisiae*, and *D. melanogaster*. Furthermore, we expanded the query set by adding the latest version of human complexes obtained from CORUM [27], and yeast complexes from SGD [28] (as of Jan. 5, 2015). Finally, as in [7, 8], we selected connected complexes of size 5 ∼25 and used them as our query networks (863 complexes in total). We assessed the performance of SEQUOIA based on the 863 real protein complexes, where 293 human complexes were searched against the fly PPI network, 289 human complexes were searched against the yeast PPI network, 141 yeast complexes were searched against the human PPI network, and 140 yeast complexes were searched against the fly PPI network. Since there are only a small number of test cases for querying fly complexes against human and yeast PPI networks, we excluded those experiments in this study.



The performance of SEQUOIA was compared against several state-of-the-art algorithms, which include: RESQUE [8], Corbi [9], NatalieQ [10], HubAlign [29], and LocalAli [30]. Although HubAlign and LocalAli are global and local network alignment algorithms, respectively, we used those algorithms to identify conserved subnetworks as network querying can be viewed as a special case of pairwise network alignment. For Corbi, we used the default parameters for the gap penalty and set the option for the query type as 1, which is for general network querying. For HubAlign, we used the default parameters (i.e., *λ*=0.1 and *α*=0.7). We also used the default parameter for NatalieQ. For LocalAli, we set the minimum number of extension (-minext) to 0 and the maximum number of extension (-maxext) to 25, since the size of the query networks ranged between 5 to 25. Default values were used for other parameters. Since LocalAli identifies multiple local complexes as its output, we selected the complex with the best score as the querying result of LocalAli.

### Performance assessment metrics

To assess various aspects of the network querying algorithms, we defined several performance metrics. First, we used the matching score to count the number of matches for each query and target species pair [31]. Given two biological complexes *Q* and *C*, the matching score is computed based on the Jaccard index between the nodes in the two biological complexes as follows:

6 $$ match\_score\left({Q,C} \right) = \frac{{\left| {{\mathcal{V}}_{Q} \cap {\mathcal{V}}_{C}} \right|}}{{\left| {{\mathcal{V}}_{Q} \cup {\mathcal{V}}_{C}} \right|}},  $$

where ${\mathcal {V}}_{X}$ is the set of nodes in the complex *X*. If the matching score is greater than the threshold, the two complexes were regarded to be a match. As in [31], we set the threshold for the matching score as 0.5. To count the number of matches, we used the known biological complexes as our gold standard reference ${\mathcal {C}} = \left \{ {C_{1},C_{2},\ldots,C_{N}} \right \}$. Given the querying result *Q*_*i*_, if there is at least one matching complex *C*_*j*_ in the gold standard reference, we counted *Q*_*i*_ as a match. Then, we report the total number of matches for each query and target species pair. That is, given the querying results ${\mathcal {Q}} = \left \{ {Q_{1},Q_{2},...,Q_{M}} \right \}$ for the *M* query complexes, we count the total number of querying results $ \left | {\left \{ {Q_{i} |match\_score\left ({Q_{i},C_{j}} \right) \ge 0.5,\forall C_{j} \in {\mathcal {C}}}, \forall Q_{i} \in {\mathcal Q} \right \}} \right |$.

Next, we defined two different types of hits that respectively measure: 1) the accuracy of the obtained querying results and 2) the capability of detecting novel functional network modules with strong biological significance. The former counts the number of querying results whose annotation is identical to the functional annotation of the query network so that it can assess the capability of a given algorithm to identify the conserved functional modules. The latter counts the number of querying results with strong biological significance, regardless of whether or not they have the same functional annotation as the query, so that it can be used to assess the ability of the network querying algorithm to predict novel potential functional modules in the target PPI network.

To evaluate the accuracy of the querying results, we picked the most significantly enriched GO term of the query network (referred to as the significant GO term). Note that the most significantly enriched GO term denotes the GO term with the lowest false discovery rate (FDR) corrected *p*-value. To this aim, we performed GO enrichment tests for the query network and the querying result. If the significant GO term in the query is also enriched in the network querying result and if its FDR corrected *p*-value is less than a threshold, we regarded the querying result as a significant hit. However, a higher number of significant hits does not necessarily imply that the network querying algorithm yields accurate results, since the querying results may potentially include a large number of functionally irrelevant proteins (i.e., proteins whose annotation does not include the significant GO term). For this reason, in order to assess the accuracy of the querying results, we additionally defined two important performance metrics: the significant specificity (SPE) and the significant functionally coherent (FC) hit. Significant SPE is defined as the relative proportion of the proteins annotated with the significant GO term among the proteins included in the querying result. Based on this definition, an accurate querying result with fewer irrelevant proteins will have a higher significant SPE. Significant FC hits were defined as hits that satisfy the following two conditions: 1) FDR corrected *p*-value should be less than a certain threshold and 2) at least 50% of the proteins included in the querying result should be annotated with the significant GO term. A network querying algorithm that can yield a larger number of significant FC hits can be viewed as being more accurate and being capable of making better predictions that are biologically more significant.

Next, in order to assess the capability of detecting novel potential functional network modules, we investigated the biological significance of the querying results. To this aim, we performed the GO enrichment test only for the querying result (i.e., not for the query network) and selected the GO term with the smallest FDR corrected *p*-value as the most significantly enriched GO term. If the FDR corrected *p*-value of the most significantly enriched GO term of the querying result is less than a threshold, we regarded the querying result as a hit. A querying result with a small FDR corrected *p*-value can be viewed as being biologically significant, even if the most significantly enriched GO term of the querying result and that of the query network do not match. As a result, for a given network querying algorithm, we can assess its capability of detecting potential functional network modules by measuring the number of hits. Furthermore, we defined the specificity as the relative proportion of proteins (in the querying result) that are annotated with the most significantly enriched GO term among all proteins included in the querying result. As before, we defined a hit as being functionally coherent (FC) – hence called a FC hit – if the FDR corrected *p*-value is less than a certain threshold and if more than 50% of the proteins in the retrieved result are annotated with the most significantly enriched GO term.

We used the latest version of GO::TermFinder [32] for the GO enrichment test, and analyzed the querying results based on three different ontology aspects: 1) cellular component (CC, GO:0005575), 2) biological process (BP, GO:0008150), and 3) molecular function (MF, GO:0003674). In the following, we mainly present the assessment results based on the ontology aspect of “cellular component”, and simulation results for other ontology aspects – i.e., “biological process” and “molecular function” – are included in the supplementary material (see Additional file 1: Section S4). The ontology and annotation files for the three species considered in our study have been downloaded from Gene Ontology Consortium [33, 34] (as of Feb. 9 2015). Then, we removed all GO terms without experimental evidence. That is, we only used GO terms having one of the following evidence codes: ‘EXP’, ‘IDA’, ‘IPI’, ‘IMP’, ‘IGI’, and ‘IEP’. Additionally, due to the hierarchical structure of GO terms, certain GO terms are annotated to a large number of proteins, where such commonly appearing GO terms would not be very informative. In order to use the GO terms that are informative, we computed the information content (IC) for each GO term as recommended in [33]. IC is defined as

7 $$ IC\left (g \right) = - \log_{2} \frac{\left | g \right |}{\left | root\left (g \right) \right |},  $$

where |*g*| is the total number of proteins with the GO term *g*, and |*r* *o* *o* *t*(*g*)| is the number of proteins under the root GO term of the GO term *g*. Note that there are three root GO terms: cellular component (CC, GO:0005575), biological process (BP, GO:0008150), and molecular function (MF, GO:0003674). In this study, we only used the GO terms whose information content is at least 2.

### Comparison of the querying results to the gold standard reference sets

**Fig. 2:**
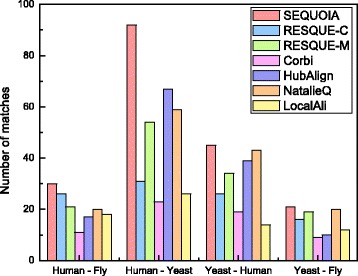
Number of matches for each query and target species pair (i.e., query species – target species)

Figure 2 shows the number of matches for each query-target species pair. The figure shows that SEQUOIA yields the largest number of matches among all tested algorithms for all query-target pairs. When querying human complexes against the fly and the yeast PPI networks, SEQUOIA clearly outperforms other methods. When querying yeast complexes against the human and the fly PPI networks, NatalieQ shows comparable performance to SEQUOIA, although SEQUOIA still yields a larger number of matches compared to all other methods. Overall, SEQUOIA resulted in 188 matches, which is almost 32 percent more compared to the number of matches achieved by the next best algorithm, NatalieQ.

### Assessing the accuracy of the network querying results

**Fig. 3:**
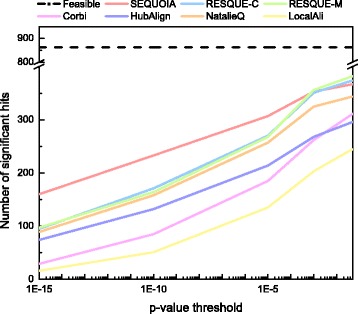
Number of significant hits for the 863 query complexes

**Fig. 4:**
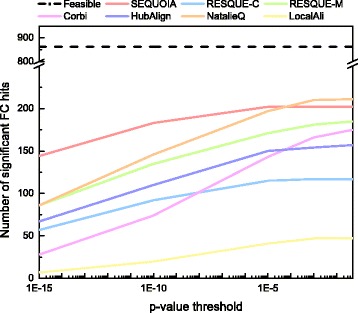
Number of significant functionally coherent (FC) hits for the 863 query complexes

Figures 3 and 4 shows the number of significant hits and significant FC hits for all 863 querying results. As we can see in Fig. 3, SEQUOIA yields a larger number of significant hits compared to other algorithms. This means that SEQUOIA can more accurately identify conserved functional network modules with the significant GO term, (i.e., the most significantly enriched GO term in the query network). RESQUE family yielded similar number of significant hits at the *p*-value threshold of 0.05, but SEQUOIA outperformed both RESQUE-C and RESQUE-M when a smaller *p*-value threshold was used. Except for SEQUOIA and RESQUE-C, the number of nodes in the querying result is generally smaller than that in the query network for other tested algorithms. As a consequence, many algorithms may fail to identify inserted nodes and yield fewer significant hits.

Figure 4 shows that SEQUOIA yields a larger number of significant FC hits compared to other algorithms. This implies that SEQUOIA produces more accurate querying results that are functionally more coherent. Compared to SEQUOIA, the number of significant FC hits for Corbi decreases quickly as the *p*-value threshold decreases. Interestingly, although RESQUE family shows similar performance in terms of the number of significant hits, the number of significant FC hits for RESQUE-C is much smaller than RESQUE-M. This result shows that using a more sophisticated method to predict the best matching subnetwork would be needed to obtain better querying results that are functionally more coherent. In fact, RESQUE-C uses a relatively simple approach to find the best matching subnetwork, which is to find the largest connected subnetwork in the reduced target network, and this may increase the chances of including a larger number of functionally irrelevant nodes in the final querying result. SEQUOIA results in higher significant hits as well as higher significant FC hits by minimizing the network conductance of the matching subnetwork and filtering out potentially irrelevant nodes based on the extension reward score. Detailed querying results for different query and target species pairs can be found in the supplementary material (see Additional file 1: Section S4).

The number of identified nodes included in the querying results and the number of nodes annotated with the most significant GO term are summarized in Table 1. The table shows that NatalieQ and RESQUE-M achieve higher significant SPE compared to SEQUOIA, but it should be noted that SEQUOIA can identify a much larger number of “annotated nodes” while keeping relatively higher significant SPE compared to other algorithms. The total number of identified nodes is comparable for SEQUOIA and RESQUE-C, although SEQUOIA results in a much higher significant SPE compared to RESQUE-C. From the perspective of potential knowledge transfer from a well-studied species to a less-studied species, the ability to achieve higher significant SPE is critical, as it implies that the network querying algorithm may be able to annotate the proteins in the querying result more accurately.

**Table 1:** Significant SPE for the ontology aspect of “cellular component”

	Identified nodes	Annotated nodes^a^	Significant SPE
SEQUOIA	9537	2568	0.269
RESQUE-C	10,213	2115	0.207
RESQUE-M	7000	1941	0.277
Corbi	4761	1149	0.241
HubAlign	7342	1526	0.208
NatalieQ	5452	1745	0.320
LocalAli	6220	892	0.143

^a^Annotation corresponding to the most significantly enriched GO term in the query network

### Capability of detecting novel functional network modules

**Fig. 5:**
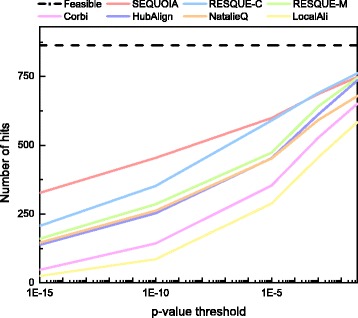
Number of hits for querying 863 biological complexes

**Fig. 6:**
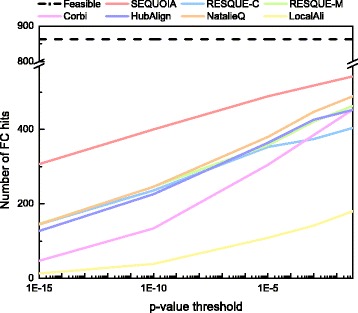
Number of functionally coherent (FC) hits for querying 863 biological complexes

Figures 5 and 6 shows the number of hits and the number of FC hits for various FDR corrected *p*-value thresholds. Feasible hits in each figure correspond to the total number of query complexes, which is the maximum number of hits that can be achieved. As we can see in Fig. 5, SEQUOIA clearly outperforms other algorithms for various *p*-value thresholds. For example, at a *p*-value threshold of 1E-10, SEQUOIA yields 29% more hits than RESQUE-C, which is the next best algorithm. This results indicate that SEQUOIA has stronger potentials to identify novel protein complexes compared to other state-of-the-art algorithms.

Next, we compared the number of FC hits for different network querying algorithms. Figure 6 shows that SEQUOIA clearly outperforms other algorithms. For example, SEQUOIA can identify 11% more FC hits than NatalieQ at a *p*-value threshold of 0.05 and almost twice as many FC hits compared to RESQUE and NatalieQ at a *p*-value threshold of 1E-15. LocalAli and NatalieQ fail to yield querying results in some test cases (i.e., these algorithms cannot identify any protein node in the target network). LocalAli and NatalieQ may not perform robustly under certain conditions (e.g., for certain query topology), which may result in a smaller number of hits. The results in Fig. 6 show that SEQUOIA’s performance is more robust compared to many other algorithms, and that SEQUOIA can more effectively detect conserved network modules with high functional coherence.

Finally, we also evaluated the functional coherence of the querying results for each algorithm. To this aim, we selected the most significantly enriched GO term in the querying result obtained by each algorithm for each query, and compute the relative proportion of proteins annotated with the most significantly enriched GO term. The results are summarized in Table 2. With the exception of NatalieQ, SEQUOIA achieves the highest SPE compared to all other algorithms. Although NatalieQ results in the highest SPE, SEQUOIA can identify about 66% more annotated nodes (i.e., proteins annotated with the most significant GO term) compared to NatalieQ, while achieving a comparable SPE. This indicates that SEQUOIA can effectively identify a larger number of protein nodes that are functionally coherence than the other tested algorithms.

**Table 2:** SPE for the ontology aspect of “cellular component”

	Identified nodes	Annotated nodes^a^	SPE		
SEQUOIA	9537	5531	0.580		
RESQUE-C	10,213	5002	0.492		
RESQUE-M	7000	3856	0.551		
Corbi	4761	2486	0.522		
HubAlign	7342	3822	0.521		
NatalieQ	5452	3324	0.610		
LocalAli	6220	2170	0.349		

^a^Annotation corresponding to the most significantly enriched GO term in the querying result

### Computation time

**Fig. 7:**
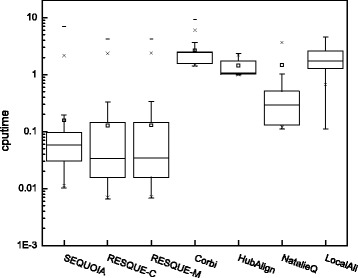
*Boxplot* of the computation time for different algorithms for the 863 queries

Figure 7 shows the box plot for the computation time for each network querying algorithm. For RESQUE, we used the MATLAB script version 1.0 and MATLAB version 2014b. Executable binaries for NatalieQ, HubAlign, and LocalAli were obtained by compiling their source code using a C++ compiler. For Corbi, we used its R package and tested the algorithm on Windows. Except for Corbi, all other algorithms were tested on Mac OS X. All computer simulations were performed on a desktop computer equipped with a 2.4 GHz Intel i7 processor and 8 GB memory. For certain queries, NatalieQ and LocalAli may require a very long time (which is significantly longer than the average computation time), and such outliers were excluded when drawing the box plot for readability. As shown in Fig. 7, the computation time of SEQUOIA is comparable to that of the RESQUE family, but it is much faster compared to other algorithms. On average, SEQUOIA yields the querying result in less than 0.06 second, and in 98% of the test cases, the algorithm needs less than a second to find the subnetwork that best matches the query.

## Conclusions

In this paper, we proposed SEQUOIA, a novel network querying algorithm that can enhance the biological significance of the query results. In order to identify conserved subnetwork regions in the target network that are similar to a given query network, the algorithm compares the two networks and estimates the node correspondence scores by using the context-sensitive random walk model. Inspired by the pair hidden Markov model that has been widely used in the comparative sequence analysis, the CSRW model effectively captures the similarities between graphs by explicitly accounting for potentially inserted/deleted nodes. Based on the estimated CSRW node correspondence scores, SEQUOIA identifies high-scoring regions (referred to as the seed networks) in the target network that bear considerable similarity with the query network. The seed network is further extended by adding neighboring nodes that reduce the network conductance of the extended network by the largest amount. This extension step identifies nearby proteins that are densely connected to other nodes in the potential network module, thereby effectively recruiting proteins that are likely to share similar functions with other proteins in the module. The final query result is obtained after pruning the matching subnetwork by removing any irrelevant nodes, thereby enhancing the separability and coherence of the identified network module. As we have shown through extensive numerical simulations based on 863 real biological complexes, our network querying algorithm SEQUOIA yields accurate query results with enhanced biological significance.

## Additional file

Additional file 1
**Section S1.** Review of the context-sensitive random walk model. This section provides detailed description of the context-sensitive random walk model. **Section S2**:Illustration of the pre-processing step. This section provides detailed description of the pre-processing step with an example. **Section S3**: Flow chart for SEQUOIA with a toy example. **Section S4**: Performance assessment for various GO ontology aspects. This section presents performance assessment results for various GO ontology aspects: cellular component, biological process, and molecular function. It also shows results for various query and target network pairs. **Section S5**: Performance improvement through post-filtering based on extension reward scores. Results in this section show the effectiveness of the pruning step based on the extension reward scores for enhancing the biological significance of the querying results. (PDF 1800 kb)
