# Supplementary material for: SEQUOIA: significance enhanced network querying through context-sensitive random walk and minimization of network conductance
Source: BMC Syst Biol. 2017 Mar 14;11(Suppl 3):20. doi: 10.1186/s12918-017-0404-6 (PMC5374659; doi:10.1186/s12918-017-0404-6)
Supplement: Additional file 1 — Section S1. Review of the context-sensitive random walk model. This section provides detailed description of the context-sensitive random walk model. Section S2:Illustration of the pre-processing step. This section provides detailed description of the pre-processing step with an example. Section S3: Flow chart for SEQUOIA with a toy example. Section S4: Performance assessment for various GO ontology aspects. This section presents performance assessment results for various GO ontology aspects: cellular component, biological process, and molecular function. It also shows results for various query and target network pairs. Section S5: Performance improvement through post-filtering based on extension reward scores. Results in this section show the effectiveness of the pruning step based on the extension reward scores for enhancing the biological significance of the querying results. (PDF 1800 kb) [file 12918_2017_404_MOESM1_ESM.pdf]

Supplementary Material for "SEQUOIA: Significance enhanced network querying through context-sensitive random walk and minimization of network conductance"

Hyundoo Jeong and Byung-Jun Yoon *

Department of Electrical and Computer Engineering, Texas A&M University, College Station, TX, USA.

∗ E-mail: bjyoon@ece.tamu.edu

S1 Review of the context-sensitive random walk model

In the context-sensitive random walk model [1], random walkers in each network to be compared can switch its mode of walk either the simultaneous walk or individual walk based on the context of the current position, *i.e.*, pair-wise node similarity of its neighboring nodes.

Figure S1 shows an example of the simultaneous walk. Note that the current position of the random walker is marked as a blue color, and pair of neighboring nodes with positive node similarity score are marked as the same color. Given the current position of the random walker (u_c, v_c), if there is a pair of neighboring nodes with positive node similarity, random walkers in each network can simultaneously move to its neighboring nodes, and its transition probability is proportional to the pair-wise node similarity score $s\left(u_i, v_j\right)$. Transition probability for the simultaneous walk is given by

$$P\big[(u_i, v_j) \,|\, (u_c, v_c)\big] = \frac{s\left(u_i, v_j\right)}{\sum_{(u_{i'}, v_{j'}) \in \mathcal{N}(u_c, v_c)} s\left(u_{i'}, v_{j'}\right)}, \tag{1}$$

where $\mathcal{N}(u_c, v_c)$ is the set of neighboring nodes for the current position of the random walker.

As shown in Figure S2, if there are no pair of neighboring nodes with positive node similarity, only one arbitrary selected random walker can move to its neighboring node so that random walker can search homologous nodes at the new position. In this case, the selection probability for the random walker is proportional to the size of the network. If the random walker in the query network is selected and it moves to its neighboring nodes, the transition probability for the individual walk is given by

$$P\big[(u_i, v_c) \,|\, (u_c, v_c)\big] = \frac{|\mathcal{U}|}{|\mathcal{U}| + |\mathcal{V}|} \times \frac{1}{|\mathcal{N}\left(u_c\right)|}, \tag{2}$$

where $|\mathcal{U}|$ and $|\mathcal{V}|$ are the size of the each network.

Otherwise, if the random walker in the target network is selected and it moves to its neighboring nodes, the transition probability for the individual walk is given by

$$P\left[(u_c, v_j)\,|\,(u_c, v_c)\right] = \frac{|\mathcal{V}|}{|\mathcal{U}| + |\mathcal{V}|} \times \frac{1}{|\mathcal{N}(v_c)|} \tag{3}$$

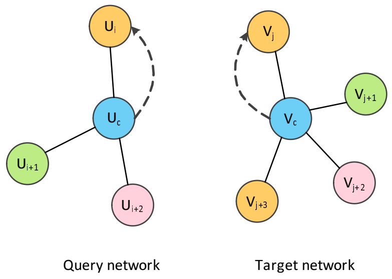

Figure S1. Illustration of the simultaneous walk over two networks.

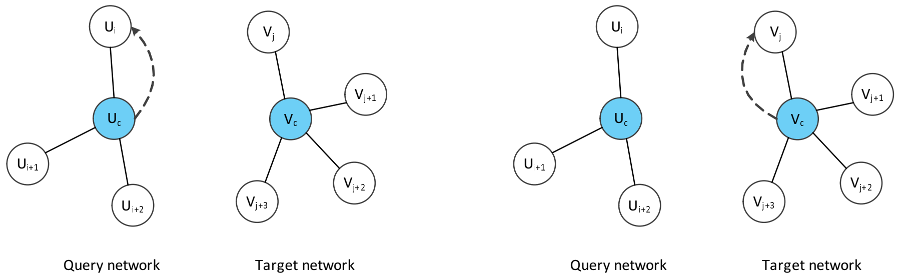

(a) Individual walk of the random walker over the query network.

(b) Individual walk of the random walker over the target network.

Figure S2. Illustration of the individual walk.

S2 Illustration of the pre-processing step

Figure S3 shows an example for removing non-homologous nodes in the target network. Non-homologous nodes in the target network are marked as a gray colored circle, and removing non-homologous nodes would make the target network disconnected as shown in Figure 3(b). Hence, we insert the pseudo edge between nodes in the disconnected subnetworks.

Figure S4 shows an example for inserting pseudo edges. Blue dotted lines represent the pairwise node similarity between the node in the query and target network, and it can be used to determine the protein homology between protein nodes in the query and target network. Even though two proteins share a common potential homologous protein in the query network, if it belongs to the same subnetwork in the target network, we will not insert the pseudo edge. Otherwise, we insert pseudo edges which are the red colored edges in Figure 4(b).

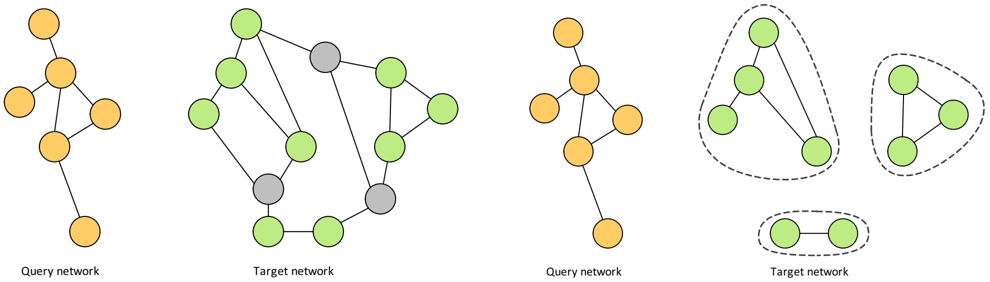

(a) Query and target network including non-homologous nodes. Gray colored nodes represent the non-homologous nodes.

(b) Ill-connected target network after removing non-homologous nodes.

Figure S3. Example for the pre-processing step: removing non-homologous nodes.

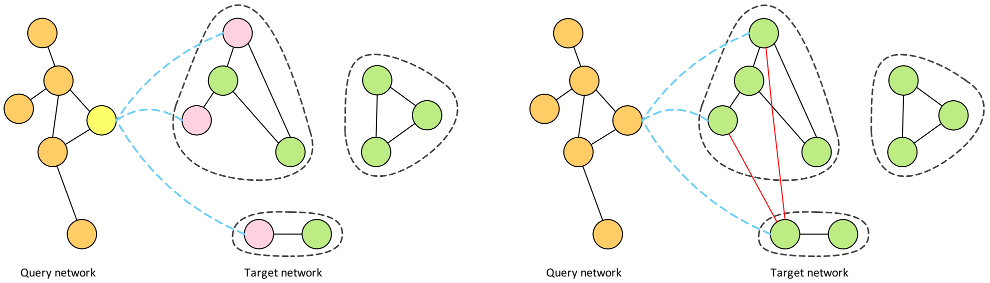

(a) Before inserting pseudo edges: pink colored proteins in the target network share the common potential homologous protein (yellow colored) in the query network.

(b) Target network with pseudo edges. Red colored edges are the inserted pseudo edges between two proteins having a common potential homologous node in the query network.

Figure S4. Example for the pre-processing step: inserting pseudo edges.

S3 Flow chart for SEQUOIA with a toy example

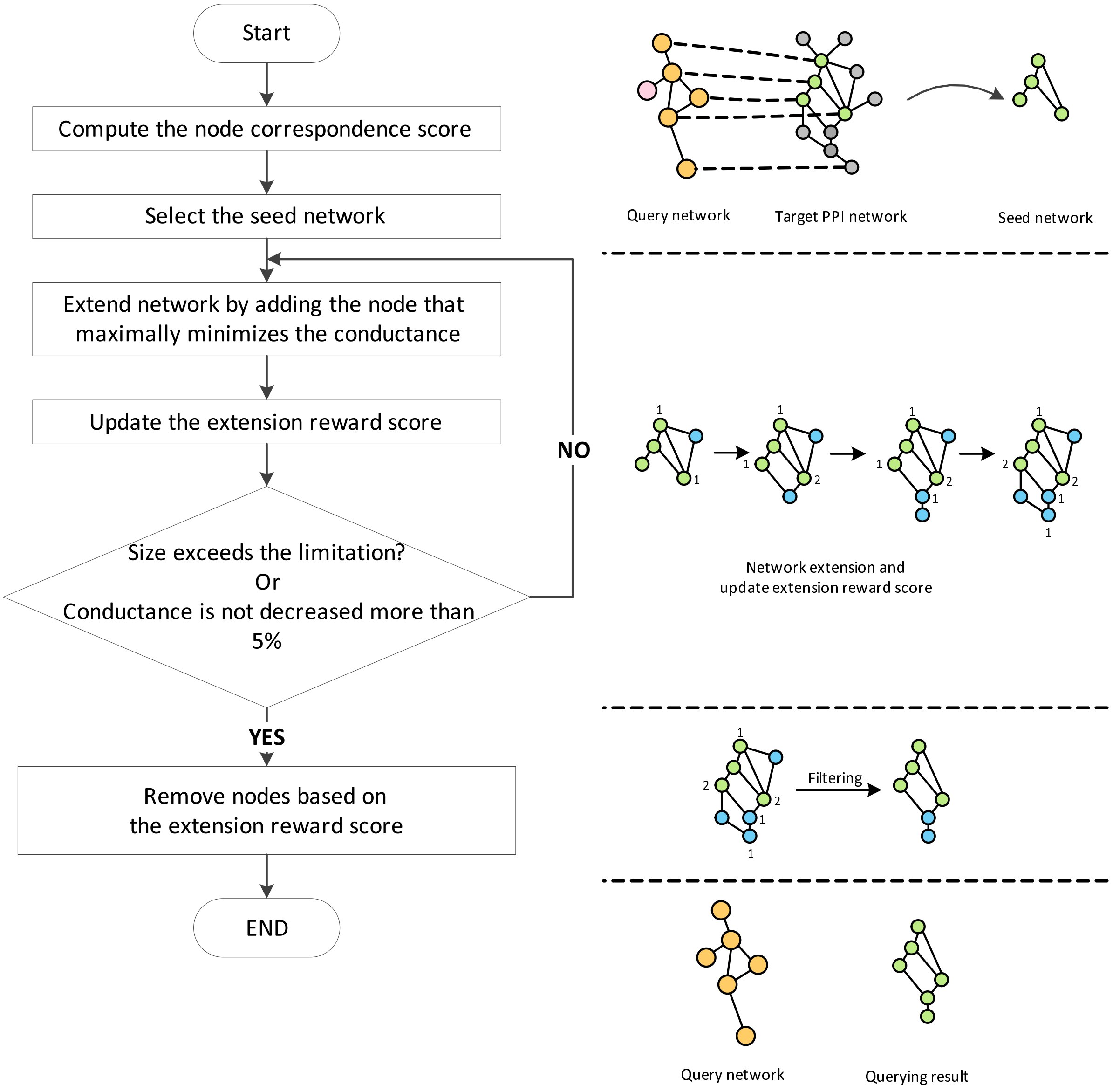

Figure S5. Flow chart of SEQUOIA and a corresponding toy example.

S4 Performance assessment for various GO ontology aspects

S4.1 Performance assessment for the ontology aspect of the cellular component

Table S1. Significant SPE for the ontology aspect of the cellular component.

	Identified nodes	Annotated nodes†	Significant SPE
SEQUOIA	9,537	**2,568**	0.269
RESQUE-C	10,213	2,115	0.207
RESQUE-M	7,000	1,941	0.277
Corbi	4,761	1,149	0.241
HubAlign	7,342	1,526	0.208
NatalieQ	5,452	1,745	**0.320**
LocalAli	6,220	892	0.143

† Annotation corresponding to the most significantly enriched GO term in the query network.

Table S2. SPE for the ontology aspect of the cellular component.

	Identified nodes	Annotated nodes‡	SPE
SEQUOIA	9,537	**5,531**	0.580
RESQUE-C	10,213	5,002	0.492
RESQUE-M	7,000	3,856	0.551
Corbi	4,761	2,486	0.522
HubAlign	7,342	3,822	0.521
NatalieQ	5,452	3,324	**0.610**
LocalAli	6,220	2,170	0.349

‡ Annotation corresponding to the most significantly enriched GO term in the querying result.

Significant hits for the ontology aspect of the cellular component.

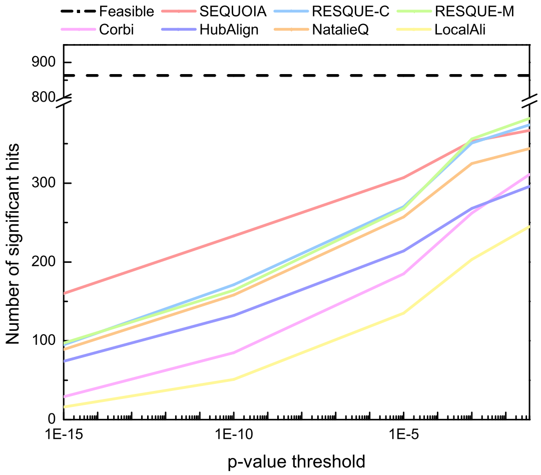

(a) Overall 863 querying results

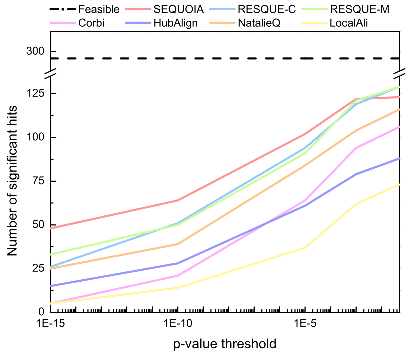

(b) Human – Fly.

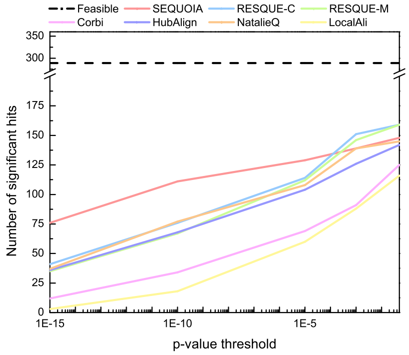

(c) Human – Yeast

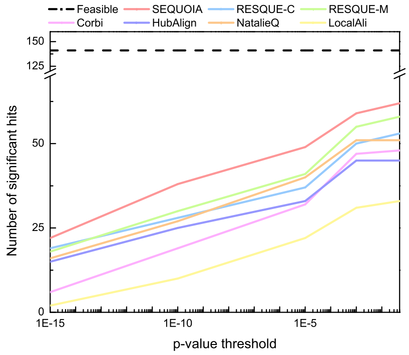

(d) Yeast – Human

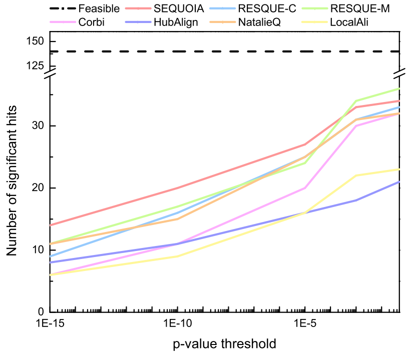

(e) Yeast – Fly

Figure S6. Number of significant hits for the ontology aspect of the cellular component: (a) overall 863 querying results, (b) querying 293 human complexes against fly PPI network, (c) querying 289 human complexes against yeast PPI network, (d) querying 141 yeast complexes against human PPI network, and (e) querying 140 yeast complexes against fly PPI network.

Significant FC hits for the ontology aspect of the cellular component.

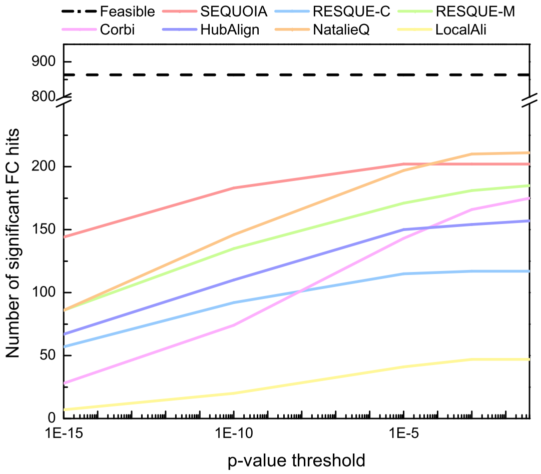

(a) Overall 863 querying results

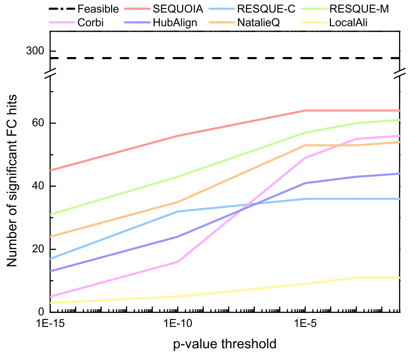

(b) Human – Fly.

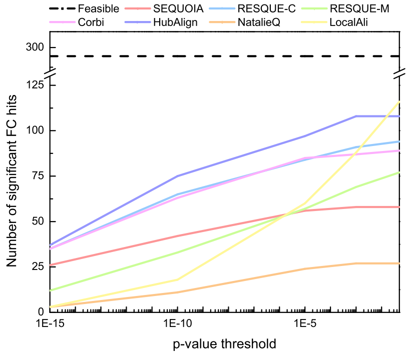

(c) Human – Yeast

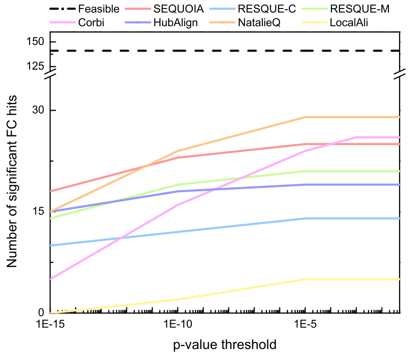

(d) Yeast – Human

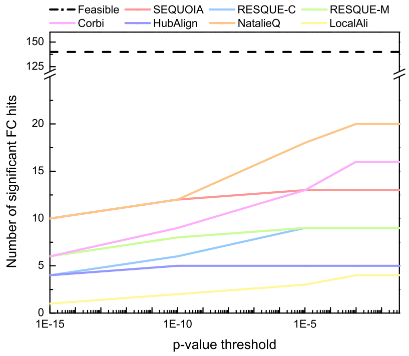

(e) Yeast – Fly

Figure S7. Number of significant FC hits for the ontology aspect of the cellular component: (a) overall 863 querying results, (b) querying 293 human complexes against fly PPI network, (c) querying 289 human complexes against yeast PPI network, (d) querying 141 yeast complexes against human PPI network, and (e) querying 140 yeast complexes against fly PPI network.

Hits for the ontology aspect of the cellular component.

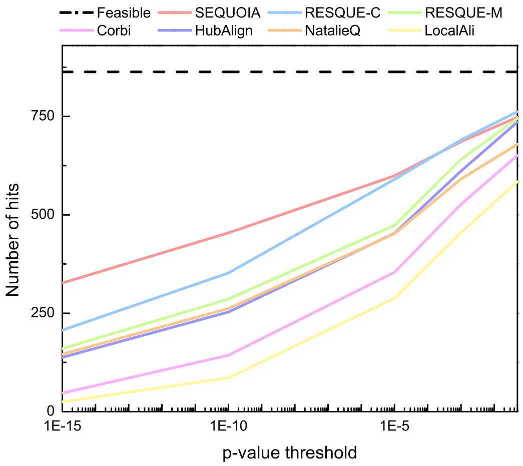

(a) Overall 863 querying results

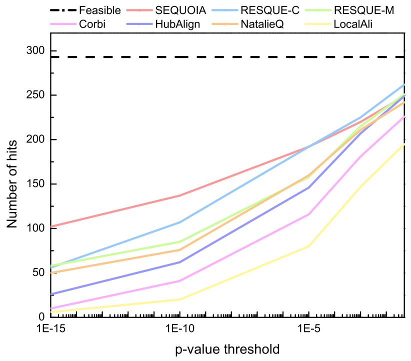

(b) Human – Fly.

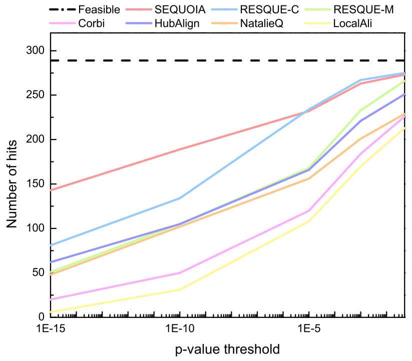

(c) Human – Yeast

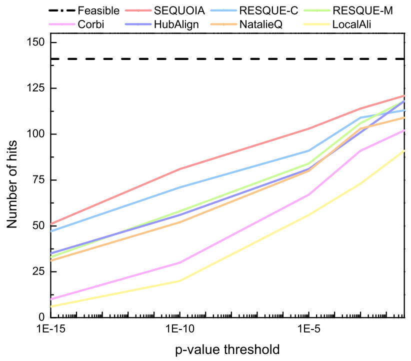

(d) Yeast – Human

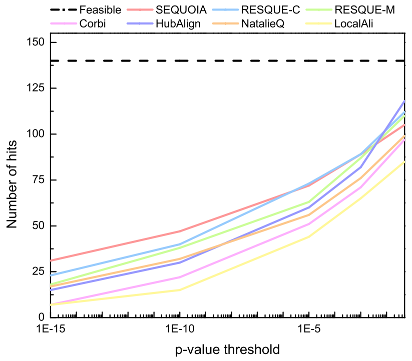

(e) Yeast – Fly

Figure S8. Number of hits for the ontology aspect of the cellular component: (a) overall 863 querying results, (b) querying 293 human complexes against fly PPI network, (c) querying 289 human complexes against yeast PPI network, (d) querying 141 yeast complexes against human PPI network, and (e) querying 140 yeast complexes against fly PPI network.

FC hits for the ontology aspect of the cellular component.

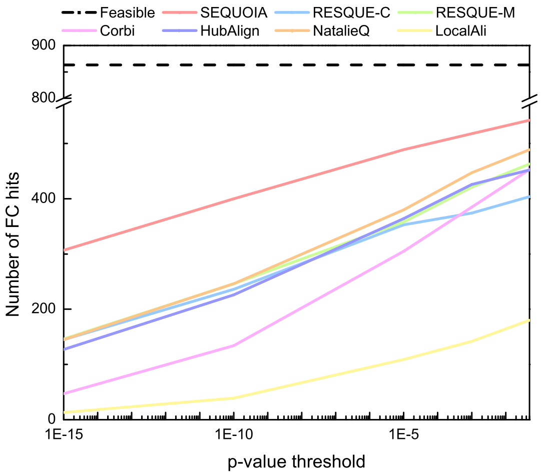

(a) Overall 863 querying results

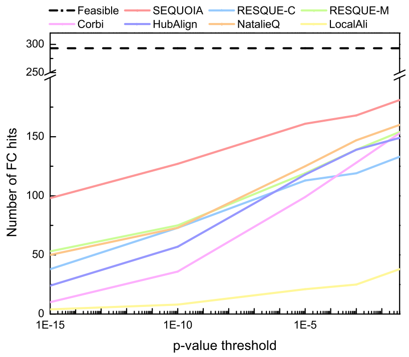

(b) Human – Fly.

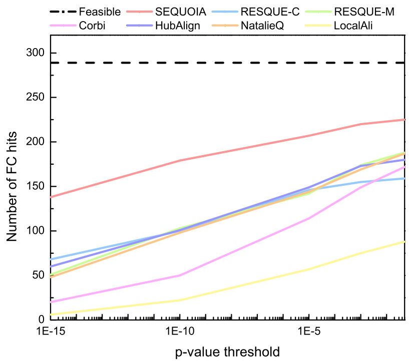

(c) Human – Yeast

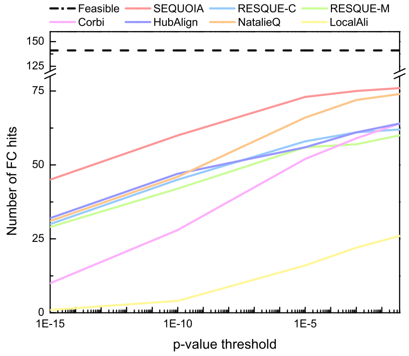

(d) Yeast – Human

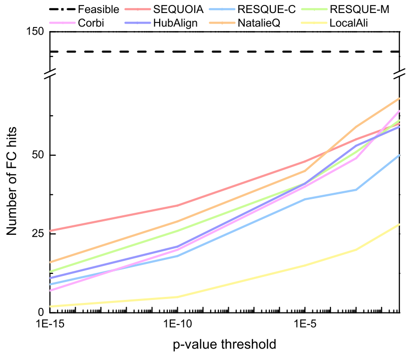

(e) Yeast – Fly

Figure S9. Number of FC hits for the ontology aspect of the cellular component: (a) overall 863 querying results, (b) querying 293 human complexes against fly PPI network, (c) querying 289 human complexes against yeast PPI network, (d) querying 141 yeast complexes against human PPI network, and (e) querying 140 yeast complexes against fly PPI network.

S4.2 Performance assessment for the ontology aspect of the biological process

Table S3. Significant SPE for the ontology aspect of the biological process.

	Identified nodes	Annotated nodes†	Significant SPE
SEQUOIA	9,537	**1,544**	0.162
RESQUE-C	10,213	1,421	0.139
RESQUE-M	7,000	1,063	0.152
Corbi	4,761	641	0.135
HubAlign	7,342	881	0.120
NatalieQ	5,452	938	**0.172**
LocalAli	6,220	480	0.077

† Annotation corresponding to the most significantly enriched GO term in the query network.

Table S4. Specificity for the ontology aspect of the biological process.

	Identified nodes	Annotated nodes†	SPE
SEQUOIA	9,537	5,168	0.542
RESQUE-C	10,213	**5,390**	0.528
RESQUE-M	7,000	3,683	0.526
Corbi	4,761	2,423	0.509
HubAlign	7,342	3,683	0.502
NatalieQ	5,452	3,097	**0.568**
LocalAli	6,220	2,235	0.359

‡ Annotation corresponding to the most significantly enriched GO term in the querying result.

Significant hits for the ontology aspect of the biological process.

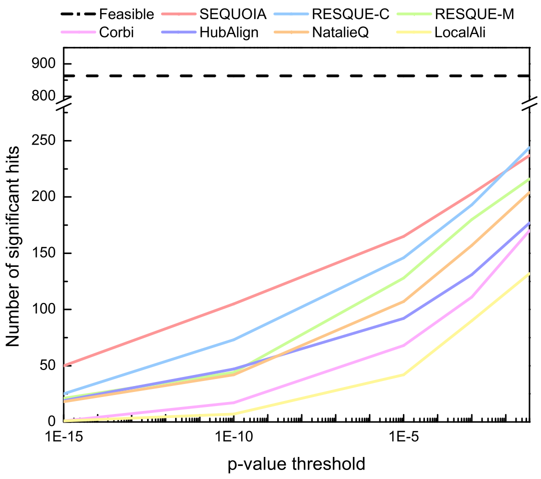

(a) Overall 863 querying results.

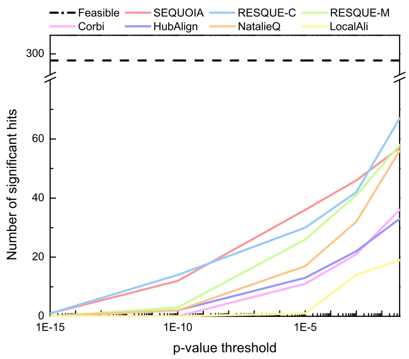

(b) Human – Fly.

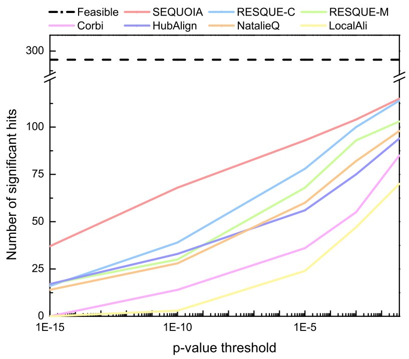

(c) Human – Yeast.

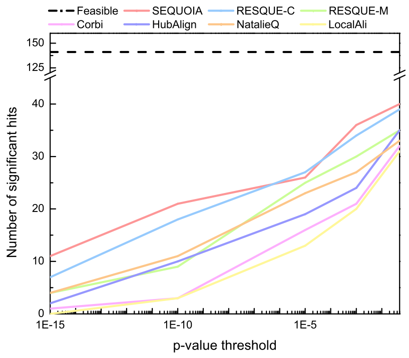

(d) Yeast – Human.

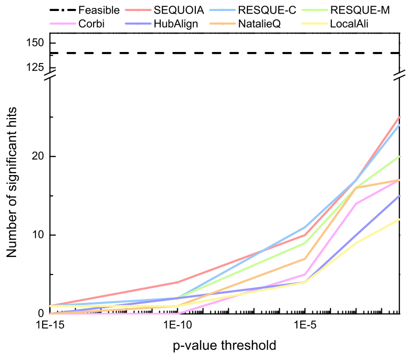

(e) Yeast – Fly.

Figure S10. Number of significant hits for the ontology aspect of the biological process: (a) overall 863 querying results, (b) querying 293 human complexes against fly PPI network, (c) querying 289 human complexes against yeast PPI network, (d) querying 141 yeast complexes against human PPI network, and (e) querying 140 yeast complexes against fly PPI network.

Significant FC hits for the ontology aspect of the biological process.

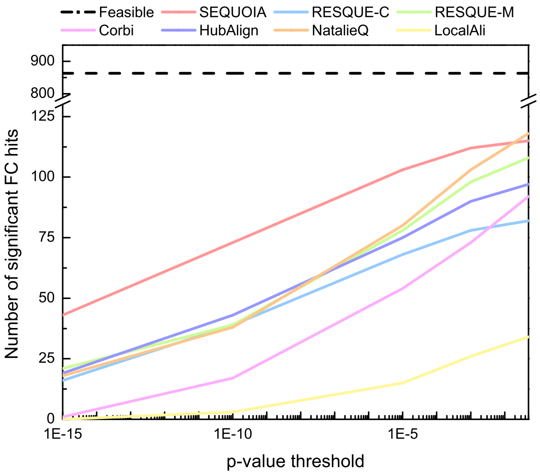

(a) Overall 863 querying results.

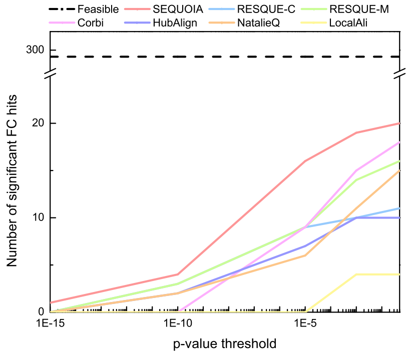

(b) Human – Fly.

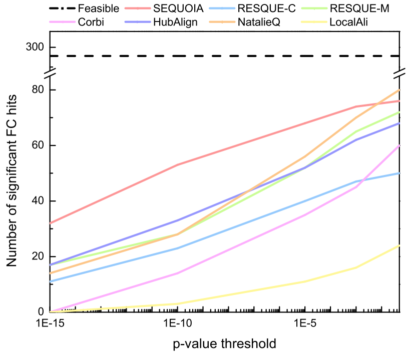

(c) Human – Yeast.

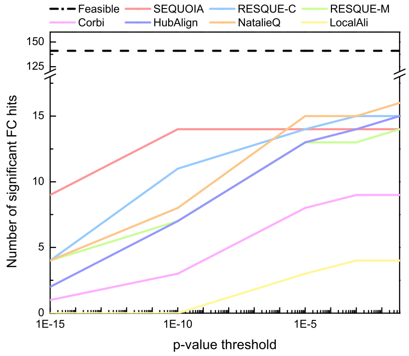

(d) Yeast – Human.

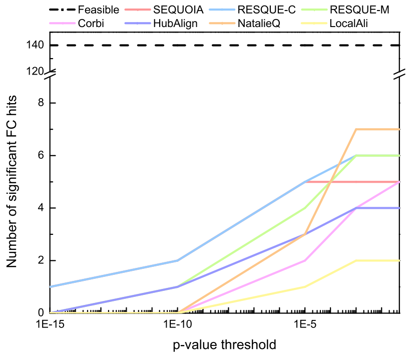

(e) Yeast – Fly.

Figure S11. Number of significant FC hits for the ontology aspect of the biological process: (a) overall 863 querying results, (b) querying 293 human complexes against fly PPI network, (c) querying 289 human complexes against yeast PPI network, (d) querying 141 yeast complexes against human PPI network, and (e) querying 140 yeast complexes against fly PPI network.

Hits for the ontology aspect of the biological process.

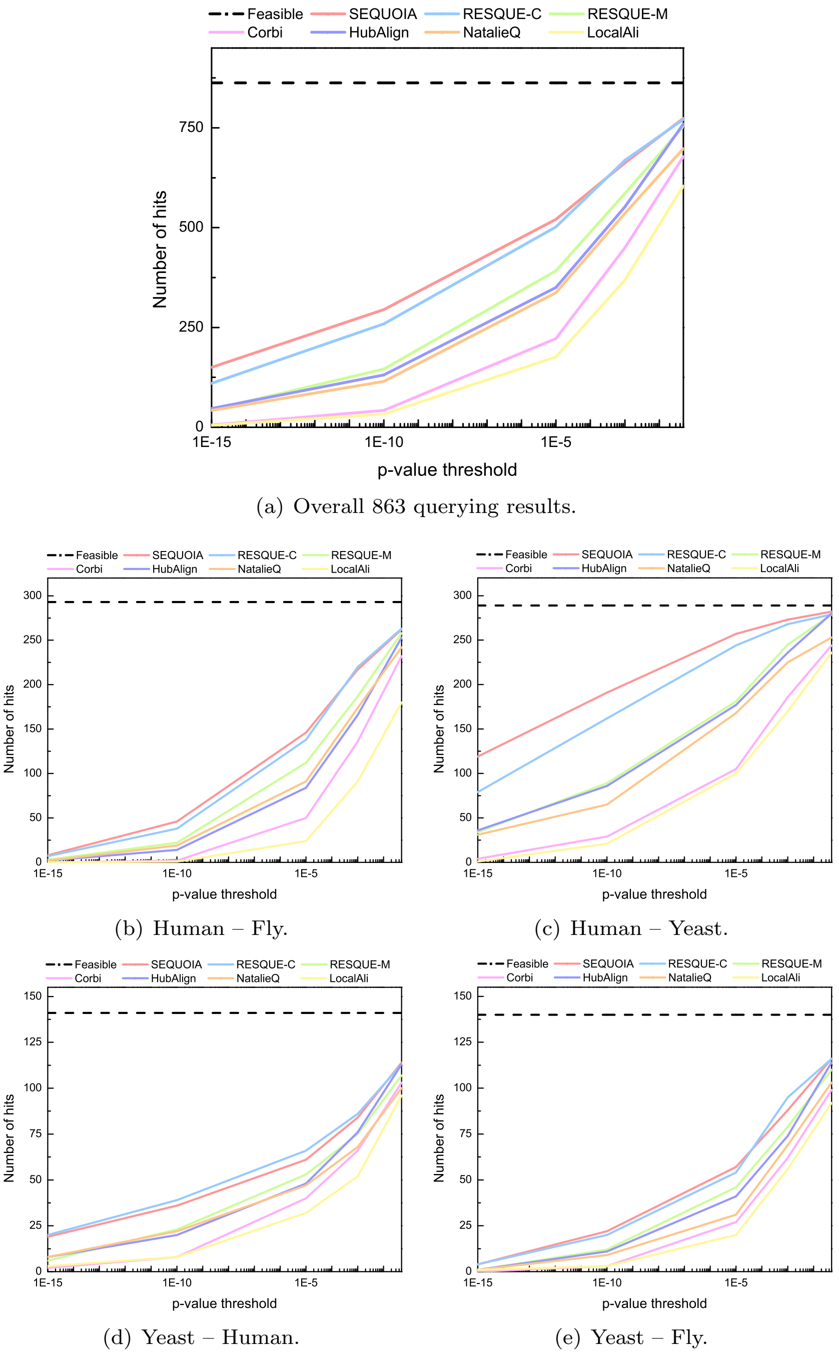

(a) Overall 863 querying results.

(b) Human – Fly.

(c) Human – Yeast.

(d) Yeast – Human.

(e) Yeast – Fly.

Figure S12. Number of hits for the ontology aspect of the biological process: (a) overall 863 querying results, (b) querying 293 human complexes against fly PPI network, (c) querying 289 human complexes against yeast PPI network, (d) querying 141 yeast complexes against human PPI network, and (e) querying 140 yeast complexes against fly PPI network.

FC hits for the ontology aspect of the biological process.

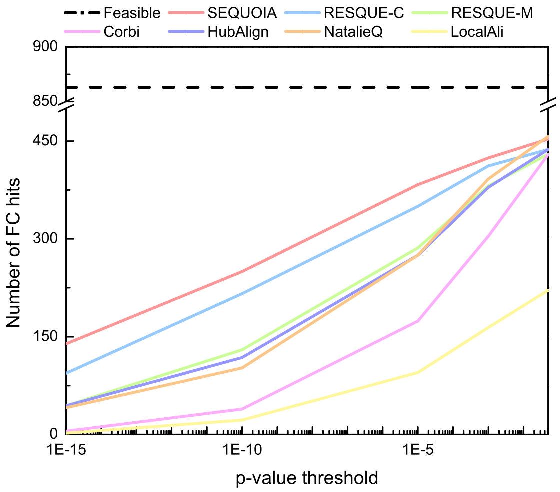

(a) Overall 863 querying results.

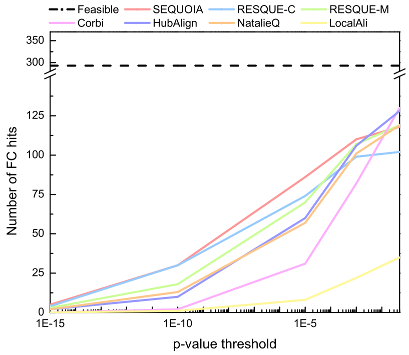

(b) Human – Fly.

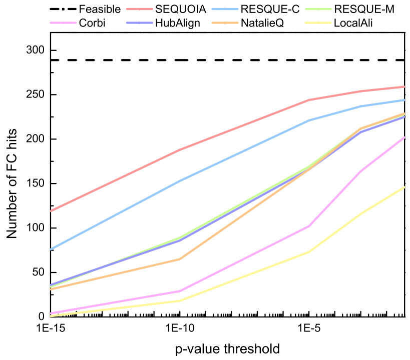

(c) Human – Yeast.

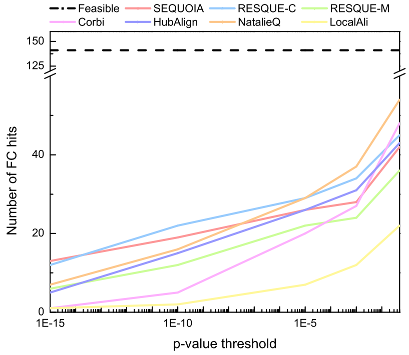

(d) Yeast – Human.

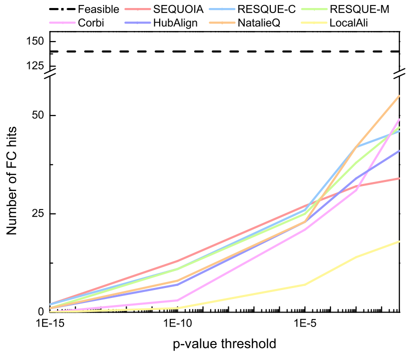

(e) Yeast – Fly.

Figure S13. Number of FC hits for the ontology aspect of the biological process: (a) overall 863 querying results, (b) querying 293 human complexes against fly PPI network, (c) querying 289 human complexes against yeast PPI network, (d) querying 141 yeast complexes against human PPI network, and (e) querying 140 yeast complexes against fly PPI network.

S4.3 Performance assessment for the ontology aspect of the molecular function

Table S5. Significant SPE for the ontology aspect of the molecular function.

	Identified nodes	Annotated nodes†	Significant SPE
SEQUOIA	9,537	**804**	0.084
RESQUE-C	10,213	869	0.085
RESQUE-M	7,000	612	0.087
Corbi	4,761	358	0.065
HubAlign	7,342	511	0.077
NatalieQ	5,452	541	**0.099**
LocalAli	6,220	368	0.059

† Annotation corresponding to the most significantly enriched GO term in the query network.

Table S6. Specificity for the ontology aspect of the molecular function.

	Identified nodes	Annotated nodes†	SPE
SEQUOIA	9,537	**4,117**	0.432
RESQUE-C	10,213	4,410	0.432
RESQUE-M	7,000	3,053	0.436
Corbi	4,761	2,004	0.421
HubAlign	7,342	2,990	0.407
NatalieQ	5,452	2,661	**0.488**
LocalAli	6,220	1,985	0.319

‡ Annotation corresponding to the most significantly enriched GO term in the querying result.

Significant hits for the ontology aspect of the molecular function.

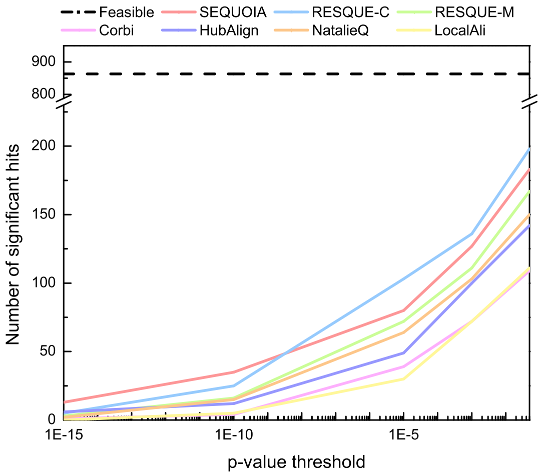

(a) Overall 863 querying results.

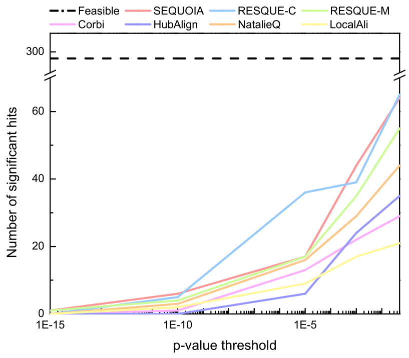

(b) Human – Fly.

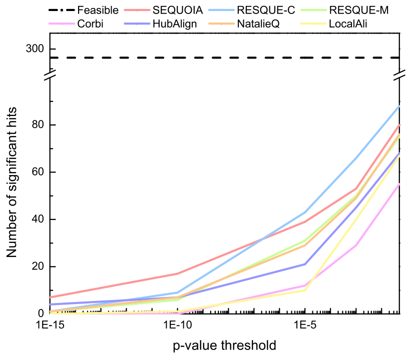

(c) Human – Yeast.

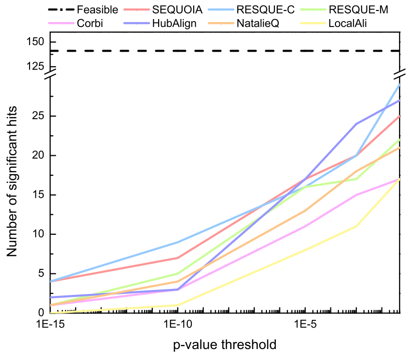

(d) Yeast – Human.

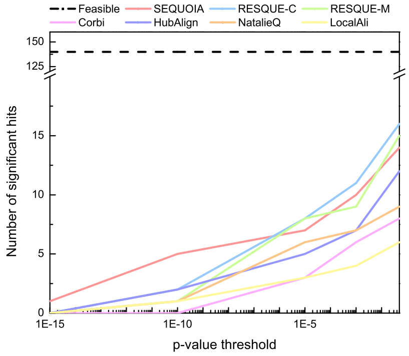

(e) Yeast – Fly.

Figure S14. Number of significant hits for the ontology aspect of the molecular function: (a) overall 863 querying results, (b) querying 293 human complexes against fly PPI network, (c) querying 289 human complexes against yeast PPI network, (d) querying 141 yeast complexes against human PPI network, and (e) querying 140 yeast complexes against fly PPI network.

Significant FC hits for the ontology aspect of the molecular function.

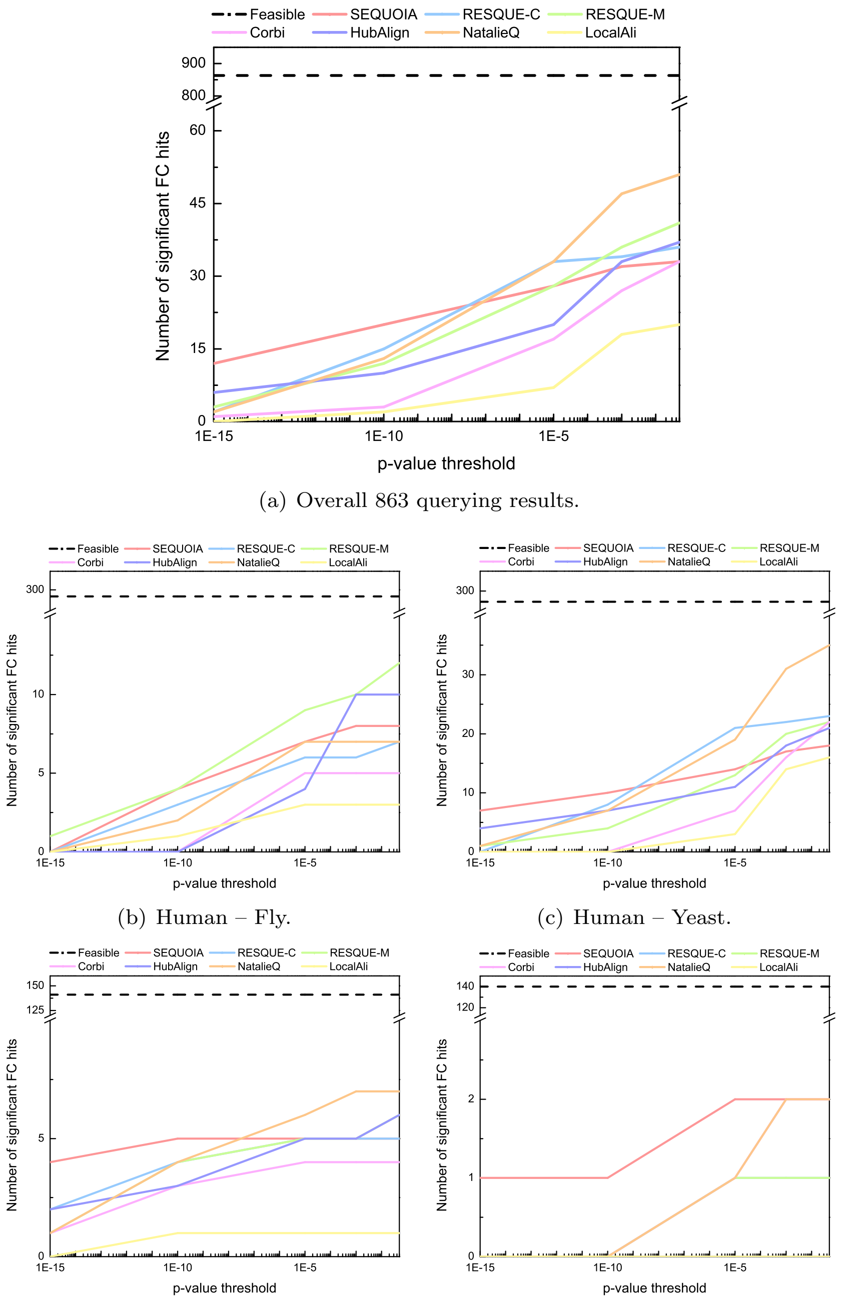

(a) Overall 863 querying results.

(b) Human – Fly.

(c) Human – Yeast.

(d) Yeast – Human.

(e) Yeast – Fly.

Figure S15. Number of significant FC hits for the ontology aspect of the molecular function: (a) overall 863 querying results, (b) querying 293 human complexes against fly PPI network, (c) querying 289 human complexes against yeast PPI network, (d) querying 141 yeast complexes against human PPI network, and (e) querying 140 yeast complexes against fly PPI network.

Hits for the ontology aspect of the molecular function.

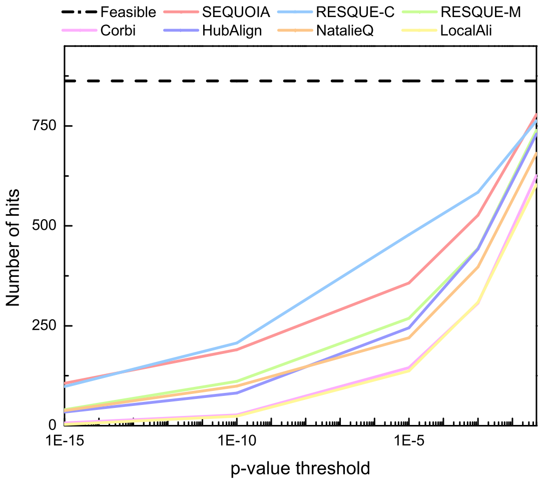

(a) Overall 863 querying results.

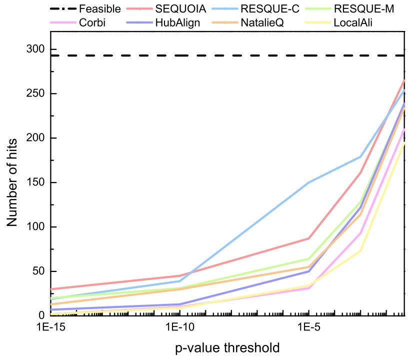

(b) Human – Fly.

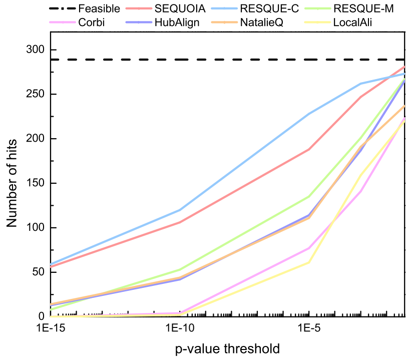

(c) Human – Yeast.

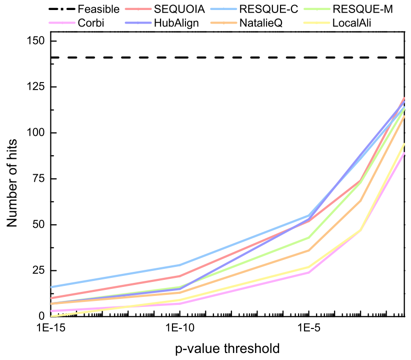

(d) Yeast – Human.

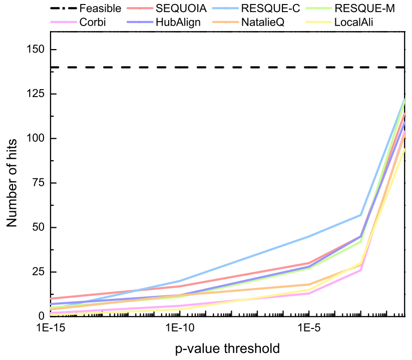

(e) Yeast – Fly.

Figure S16. Number of hits for the ontology aspect of the molecular function: (a) overall 863 querying results, (b) querying 293 human complexes against fly PPI network, (c) querying 289 human complexes against yeast PPI network, (d) querying 141 yeast complexes against human PPI network, and (e) querying 140 yeast complexes against fly PPI network.

FC hits for the ontology aspect of the molecular function.

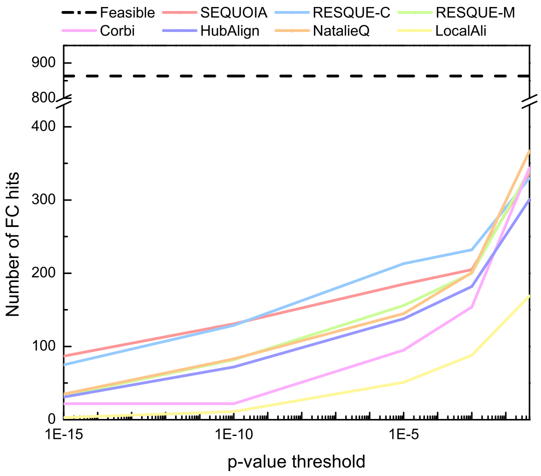

(a) Overall 863 querying results.

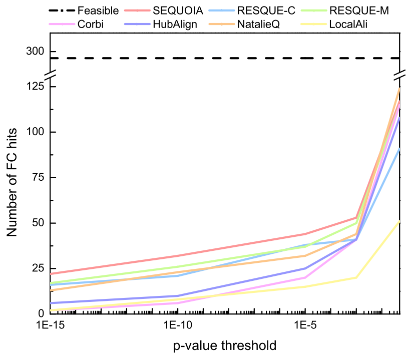

(b) Human – Fly.

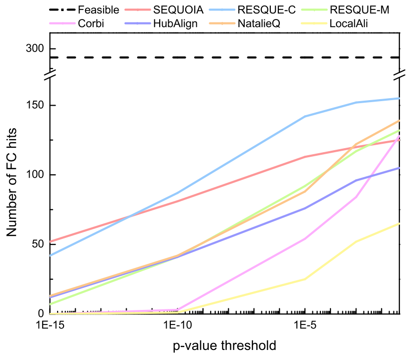

(c) Human – Yeast.

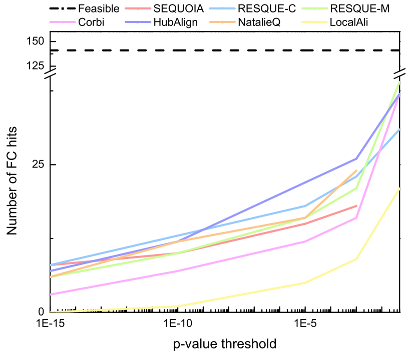

(d) Yeast – Human.

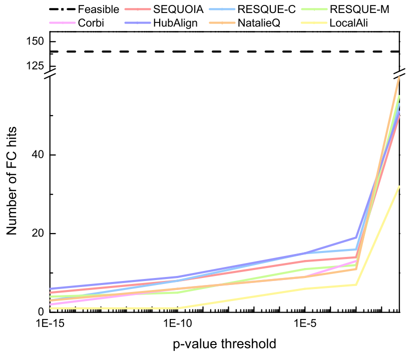

(e) Yeast – Fly.

Figure S17. Number of FC hits for the ontology aspect of the molecular function: (a) overall 863 querying results, (b) querying 293 human complexes against fly PPI network, (c) querying 289 human complexes against yeast PPI network, (d) querying 141 yeast complexes against human PPI network, and (e) querying 140 yeast complexes against fly PPI network.

S5 Performance improvement through post-filtering based on the extension reward score

After the seed network is fully extended, removing nodes based on the extension reward score can improve the biological significance of the querying result. It should be noted that, although our objective is not to find the densely connected subnetwork, SEQUOIA still identifies a dense subnetwork in the target network because i) proteins having a direct interaction are highly likely to share the common functionality [2], and ii) several know biological complexes tend to be densely connected [3–5]. To this aim, we removed some nodes in the extended network based on the extension reward score, where it can measure the relative contribution of each node to make a dense network.

Figure S18 shows the performance comparison of the querying results with and without the post-filtering based on the extension reward score. As we can see in Table S7, although the post-filtering removes about 2,100 nodes in the querying results over 863 querying cases, Figure 18(a) and Figure 18(c) show that it has a negligible effect on the significant hits and hits. However, Figure 18(b) and Figure 18(d) show that number of significant FC hits and FC hits are substantially improved by applying the post-filtering based on the extension reward score, where it also leads the enhanced significant SPE and SPE.

Table S7. Significant specificity and specificity for SEQUOIA with and without the post-filtering for the ontology aspect of the cellular component.

	Identified nodes	Annotated nodes†	Significant SPE	Annotated nodes‡	SPE
SEQUOIA with post-filtering	9,537	2,568	0.269	5,531	0.580
SEQUOIA without post-filtering	11,703	2,761	0.236	6,039	0.516

† Number of nodes having the annotation corresponding to the most significantly enriched GO term of the query network.
‡ Number of nodes having the annotation corresponding to the most significantly enriched GO term of the querying result.

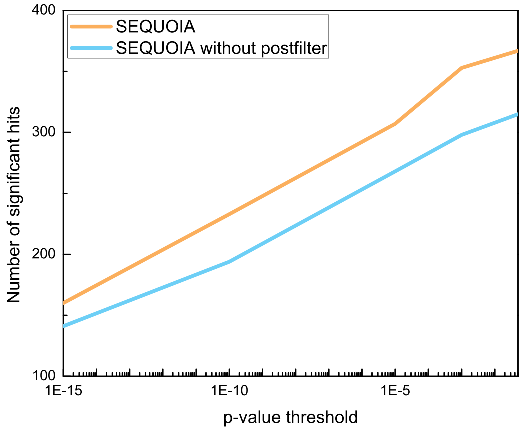

(a) Number of significant hits.

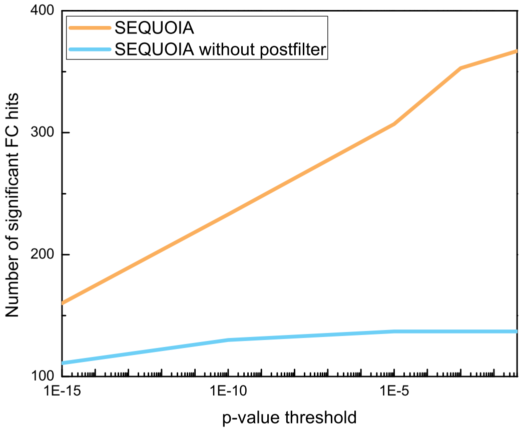

(b) Number of significant FC hits.

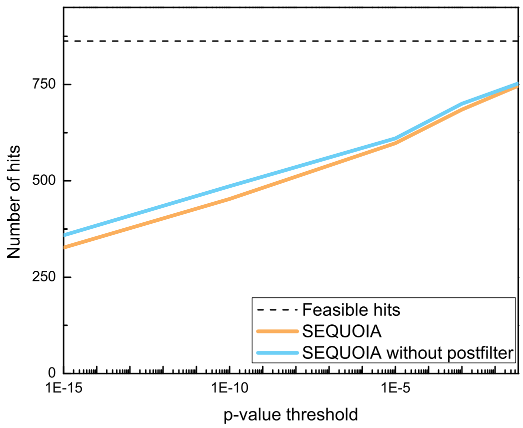

(c) Number of hits.

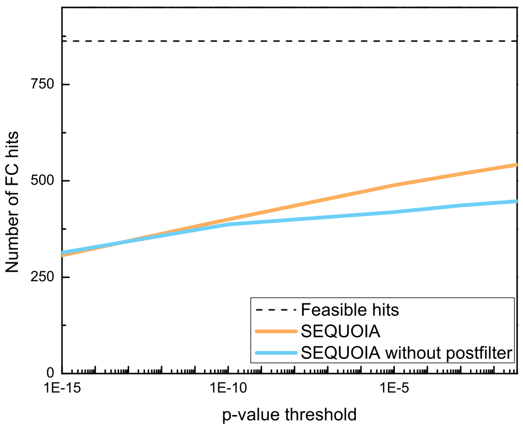

(d) Number of FC hits.

Figure S18. Performance comparison of SEQUOIA with and without the post-filtering for the ontology aspect of the cellular component.

References

1. Jeong, H., Yoon, B.-J.: Effective estimation of node-to-node correspondence between different graphs. IEEE Signal Processing Letters 22, 661–665 (2015).

2. Sharan, R., Ulitsky, I., Shamir, R.: Network-based prediction of protein function. Mol. Syst. Biol. 3, 88 (2007).

3. Girvan, M., Newman, M.E.J.: Community structure in social and biological networks. Proc. Natl. Acad. Sci. USA 99, 7821–7826 (2002).

4. Newman, M.E.J.: Finding community structure in networks using the eigenvectors of matrices. Phys. Rev. E 74, 036104 (2006).

5. Spirin, V., Mirny. L.A.: Protein complexes and functional modules in molecular networks. Proc. Natl. Acad. Sci. USA 100, 12123–12128 (2003).
